# Retraction: Prevalence of rheumatic heart disease in a major referral cardiology clinic in Ethiopia: A retrospective cross-sectional study

**DOI:** 10.1371/journal.pone.0258068

**Published:** 2021-09-23

**Authors:** Melkamu H. Asmare, Frehiwot Woldehanna, Samuel Hunegnaw, Luc Janssens, Bart Vanrumste

Following the publication of this article [1] concerns were raised by the Department of Internal Medicine, School of Medicine, Addis Ababa University. Specifically:

- In contrast to what was reported in the article, the study did not have prospective ethics approval by the research ethics committee of the Department of Internal Medicine, College of Health Sciences, Addis Ababa University.
- The data are incomplete and do not accurately represent the experimental outcomes.

In light of the concerns affecting the ethics approval for this study and the reliability of the data reported in this study, the authors retract this article.
